# Adherence to a care pathway for inflammatory bowel disease in the southwest region of the Netherlands: results of a mixed-methods implementation study

**DOI:** 10.1136/bmjoq-2025-003583

**Published:** 2025-10-22

**Authors:** Elyke Hinke Visser, Sanne Allers, Reinier C A van Linschoten, Alexander G L Bodelier, Claire Fitzpatrick, Vincent de Jonge, Hestia Vermeulen, Evelyne Verweij, Sanne K van der Wiel, Daniëlle van der Horst, Christine Janneke van der Woude, Desiree van Noord, Rachel Louise West

**Affiliations:** 1 Department of Gastroenterology & Hepatology, Franciscus Hospital, Rotterdam, Zuid-Holland, The Netherlands; 2 Department of Gastroenterology & Hepatology, Erasmus MC, Rotterdam, Zuid-Holland, The Netherlands; 3 Department of Health Policy & Management, Erasmus University Rotterdam, Rotterdam, The Netherlands; 4 Department of General Practice, Erasmus MC, Rotterdam, Zuid-Holland, The Netherlands; 5 Department of Gastroenterology & Hepatology, Amphia Hospital, Breda, The Netherlands; 6 Department of Gastroenterology & Hepatology, IJsselland Hospital, Capelle aan den IJssel, The Netherlands; 7 Department of Gastroenterology & Hepatology, Albert Schweitzer Hospital, Dordrecht, The Netherlands; 8 Department of Gastroenterology & Hepatology, Ikazia Hospital, Rotterdam, The Netherlands; 9 Department of Gastroenterology & Hepatology, Maasstad Hospital, Rotterdam, The Netherlands; 10 Department of Gastroenterology & Hepatology, Reinier de Graaf Gasthuis, Delft, The Netherlands; 11 Crohn & Colitis NL, Woerden, The Netherlands; 12 Department of Healthcare Related Education, Radboud University Nijmegen, Nijmegen, The Netherlands

**Keywords:** Chronic disease management, Clinical practice guidelines, Compliance, Implementation science, Quality improvement

## Abstract

**Background:**

In southwest Netherlands, hospitals collaborate to provide high-quality care for inflammatory bowel disease (IBD). To achieve this, a care pathway (CP) was implemented for treating IBD with advanced therapies. This study assessed the adherence to the CP and identified implementation barriers and facilitators.

**Methods:**

A mixed-methods study was conducted. Quantitative data collected from health records from December 2020 to March 2023 were used to evaluate adherence, and differences were analysed with generalised mixed models. Surveys and semistructured interviews with healthcare providers (HCPs) were used to identify barriers and facilitators, using the extended normalisation theory.

**Results:**

The study included 299 patients. Documentation of repeated screening for infectious diseases when prior tests exceeded 1 year decreased (p<0.001). Adherence to ordering blood tests as advised increased (p<0.001). For patients experiencing a flare, a small but significant increase was observed in the use of validated questionnaires for scoring disease activity (p=0.004). Adherence improved in registering smoking status (p=0.003), side effects (p<0.001), medication adherence (p<0.001) and ordering advised blood tests as recommended (p<0.001). Weight registration decreased (p=0.002).

From 85 surveys, 42 were completed, with 11 interviews conducted. Facilitators were improving collaboration and the potential to standardise care. Barriers were the complexity of the implementation in health records, the difficulty for providers to change routines and IBD heterogeneity.

**Conclusions:**

Adherence to the CP appears to be challenging, due to the difficulty HCPs experience in changing routines. Discrepancies between performed and documented tasks may affect adherence rates. The gradual improvement suggests increased familiarity with the CP may enhance adoption.

**Trial registration:**

MEC-2020-075.

**WHAT IS ALREADY KNOWN ON THIS TOPIC**

- Care pathways (CPs) are used to standardise treatment and improve outcomes in chronic diseases such as inflammatory bowel disease; however, evidence on real-world adherence and implementation is limited.

**WHAT THIS STUDY ADDS**

- The longitudinal analysis showed a gradual improvement in adherence over time and identified barriers such as challenges with the use of the CP in electronic health records, facilitators such as improved collaboration.

**HOW THIS STUDY MIGHT AFFECT RESEARCH, PRACTICE OR POLICY**

- This study can help inform clinical practices to improve uptake of CPs and support policy decisions aimed at standardising care.

## Background

 Inflammatory bowel disease (IBD) is a chronic inflammatory disease of the gastrointestinal tract. Patients can experience severe intestinal symptoms, such as diarrhoea and abdominal pain, as well as extraintestinal symptoms, such as joint and eye inflammation.^1^,^3^ IBD is often diagnosed at a young age, and due to the chronic relapsing course of the disease, many patients need long-term treatment. In recent years, there has been a rapid evolution in the development of medical treatment strategies. The complexity of the disease, combined with this development, has led to many different and costly treatment approaches.^4^

The range of new treatment strategies introduces variability in care between healthcare providers (HCPs).^5^ Variability refers to differences in diagnostic approaches, treatment choices, monitoring strategies and adherence to clinical guidelines.^6 7^ While some variability may be explained by differences in patient populations, it reflects differences in HCPs’ experience and expertise. HCPs specialised in IBD or those working in academic settings tend to have higher adherence to clinical guidelines, which is associated with improved outcomes.^6 7^

To reduce unwarranted variability and improve outcomes, quality improvement (QI) projects have been conducted in IBD care.^8^ These initiatives include education (eg, vaccination safety), implementation of screening algorithms (eg, iron deficiency) and use of electronic decision-support tools.^9^,^11^ A previsit planning initiative has been associated with increased clinical remission rates, highlighting the potential of QI projects to improve patient outcomes.^12^ Care pathways (CP) are also considered QI projects. CPs are care practices that are based on guidelines and evidence where available. Adherence to CPs helps to ensure that patients receive care that is based on the most up-to-date evidence and is consistent with the highest standards of quality and safety of care. CPs can reduce variability as HCPs follow the same treatment protocols and enhance quality of care, optimise resource utilisation and reduce healthcare costs.^13^,^16^

An essential component in the evaluation of QI projects, such as CPs, is the assessment of the implementation process and adherence to the intervention, as this indicates the usefulness of the intervention. Additionally, without a comprehensive understanding of which aspects were delivered and the barriers and facilitators, it is not possible to accurately assess the effectiveness.^17^ This study aimed to assess adherence to a CP for patients with IBD treated with advanced therapies and determine barriers and facilitators for the development and implementation.

## Methods

### The IBD Value Study

This study is part of a broader scientific initiative known as IBD Value, conducted in eight hospitals in the southwest of the Netherlands. These hospitals have collaborated since 2015 to deliver high-quality and uniform care throughout the region. To facilitate this, IBD Value was designed. Initially, a baseline year was conducted to assess the standard of care and collect outcomes as defined by the standard set of the International Consortium for Health Outcomes Measurement from December 2020 to December 2021.^18^ Subsequently, during a 3-month implementation period (December 2021–March 2023), a CP was developed and implemented for the treatment of IBD using biologics and small molecules. The CP was implemented in six hospitals, while the remaining two hospitals served as controls. Following the implementation period, outcomes were reassessed over a subsequent 12-month period (March 2022–March 2023).

Eligible patients for the IBD Value Study were adult patients with a clinician-confirmed IBD diagnosis for at least 3 months and were being treated with advanced therapies in one of the participating centres. Patients were excluded if they did not have an adequate understanding of the Dutch language or if they were not able to access the internet to fill in questionnaires. An overview of the development and implementation of the CP can be found in our published protocol.^19^

### Care pathway (CP)

The CP is divided into protocols, each offering recommendations on a specific aspect of IBD treatment with advanced therapies. For each registered biologic or small molecule, a protocol was developed that advises on steps before initiating medication, timing of outpatient visits and additional testing requirements during follow-up. The protocol used for this process is referred to as the *medication induction protocol*. Following 1 year of treatment, patients transition to the *maintenance protocol*. This protocol delineates the schedule for outpatient visits and the diagnostic tests to be conducted. When patients experience a worsening of symptoms during their treatment, HCPs can use the *flare protocol*, which provides guidelines for the management of suspected exacerbations of the disease. Furthermore, the CP includes a protocol with recommendations for an annual outpatient visit (*annual outpatient visit protocol),* specifying key consultation questions, components of the physical examination to be documented, minimum required blood tests and the use of validated questionnaires for assessing disease activity, such as the Harvey Bradshaw Index, Simple Clinical Colitis Activity Index and Crohn’s Disease Activity Index.

### Study design

To provide a comprehensive insight into the implementation and adherence to the CP, a mixed-methods study was conducted. To assess adherence to the CP, quantitative data were collected from Electronic Health Records (EHRs) for both the pre-implementation (December 2020–December 2021) and post implementation period (March 2022–March 2023). Data were collected for both periods to determine whether HCPs were following the CP or if they were already adhering to the same steps during the pre-implementation period. The post implementation period was divided into two 6-month intervals (6 months and 12 months post implementation) to assess potential changes in adherence. Interviews were conducted in September 2022, 6 months following the end of the implementation period. Questionnaires were also sent in September 2022, with a reminder 2 weeks thereafter. Both methods were used to identify the barriers and facilitators associated with the implementation of the CP.

### Patient sampling

For this study, we randomly sampled approximately 25% of those included in the IBD Value Study and were treated in the intervention hospitals.

### Data collection and measures

#### Quantitative evaluation

Given the structure of the CP in protocols, adherence was evaluated per protocol and per period (pre-implementation, 6 months post implementation and 12 months post implementation). For each patient, we determined their current phase and subsequently evaluated adherence to the corresponding protocol. For components involving blood tests or outpatient visits, it was evaluated whether this was done as advised, whether more or less tests or visits were done and whether the HCP registered a reason for deviation. For the remaining components of the protocols, the EHR was examined to determine whether a registration was found. Adherence was defined per component as the number of times an action was registered divided by the number of times the protocol was applicable. Differences in adherence outcomes between periods were analysed with generalised mixed models. Each outcome variable was modelled as a function of the measurement period as a fixed effect. To account for the repeated measurement and the hierarchical structure of the data, a random intercept was included for each participant. All analyses were conducted in R.^20^

#### Qualitative evaluation

To assess facilitators and barriers for implementing the CP, the Barriers and Facilitators Assessment Instrument (BFAI) and Normalisation Measure Development Questionnaire were used. Both are validated questionnaires to determine barriers and facilitators for implementing an intervention and to evaluate the implementation progress.^21^,^23^ In the case of overlap between questions, the respective question from the BFAI was used. Questions specific to barriers and facilitators of CPs and open-ended questions were added to the survey. The questionnaires were sent digitally to all HCPs treating patients with IBD and supporting staff in the intervention hospitals.

In addition to the questionnaires, semistructured interviews on the development and implementation process were conducted with HCPs. A topic list was used to guide the interviews. Interviews were audio recorded and transcribed by one member of the study team (SA) and another member (EHV) coded the interviews using an inductive thematic analysis in the themes of barriers and facilitators. This process was repeated until no new concepts were identified. Trustworthiness was ensured through the discussion of the results by the authors.^24^

Qualitative data from both the questionnaires and interviews were summarised using the extended Normalisation Process Theory (eNPT) as a theoretical framework. The eNPT identifies factors that facilitate or inhibit the implementation of an intervention, such as the CP. This theory identifies four core constructs that can be used to explain the normalisation of an intervention: capability, capacity, potential and contribution. The first construct, capability, suggests that an intervention is likely to become normalised if its elements are effectively applied and integrated into daily practice. The second construct, capacity, is defined as the resources available to implement the CP. The construct potential encompasses users’ individual intentions and commitments. The final construct, contribution, reflects users’ investments in developing and implementing the intervention.^25^,^28^

### Patient and public involvement

Crohn & Colitis NL (Dutch Crohn’s and Colitis Patient Organisation) collaborated in the design of the IBD Value Study. They critically revised the study and helped in piloting the questionnaires. They were involved in the working group that was responsible for the development of the CP.

## Results

In the IBD Value Study, 841 patients from the intervention hospitals were included, of which 299 were randomly selected for this study. During the pre-implementation period, adherence to the medication induction protocol was assessed 67 times, while 288 maintenance visits and 98 suspected flares were evaluated. Regarding the annual outpatient visit protocol, adherence was evaluated for 215 visits.

Adherence to the medication induction protocol was assessed 42 times during the first 6 months after implementing the CP. A total of 290 maintenance visits, 108 suspected flares and 230 annual outpatient visits were evaluated.

In the 12 months post implementation, adherence to the medication induction protocol was assessed six times. During this period, a total of 255 maintenance visits, 30 suspected flares and 113 annual outpatient visits were evaluated.

Statistically significant differences between the pre-implementation period, 6 months post implementation and 12 months post implementation per protocol are presented; detailed results of the adherence per protocol are available in online supplemental table 1.

### Adherence

#### Medication induction protocol

**Figure 1:**
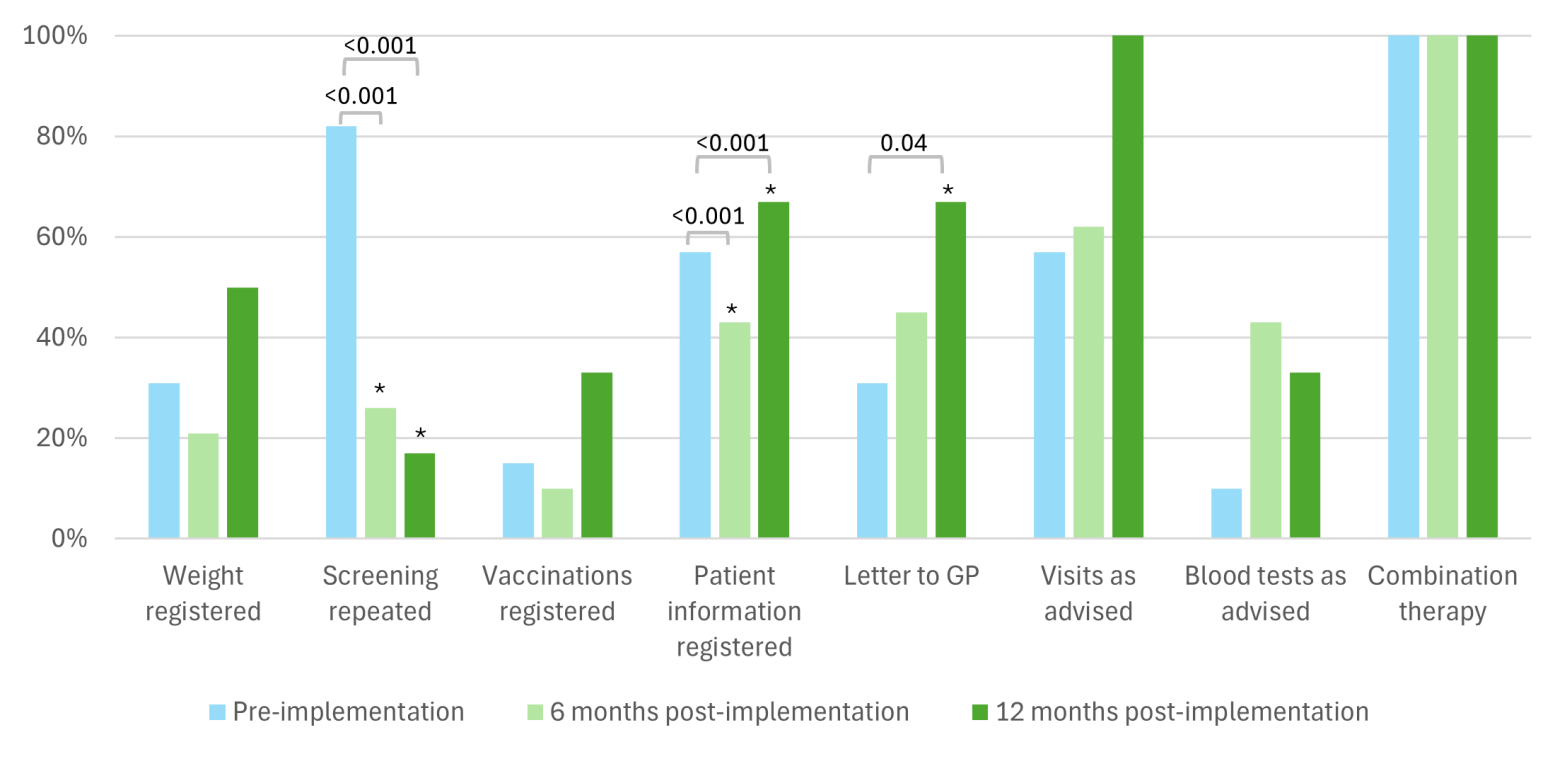
Adherence medication induction protocol. *Significant difference compared with the pre-implementation period. GP, general practitioner.

Adherence to the induction protocol yielded mixed results (figure 1). Registered repeated screenings for HIV, tuberculosis and hepatitis B and C among patients last screened over a year ago decreased significantly, from 82% pre-implementation to 26% at 6 months and 17% at 12 months post implementation (p<0.001 for both). Documentation of explanations for newly initiated medication decreased at 6 months (43%, p<0.001), but increased at 12 months post implementation (67%, p<0.001). Sending informative letters to general practitioners regarding medication increased from 31% pre-implementation to 67% at 12 months post implementation (p=0.04). Scheduling outpatient visits as recommended or documenting deviations improved from 57% pre-implementation to 62% at 6 months, reaching 100% at 12 months post implementation; formal statistical analysis was not performed due to complete adherence.

#### Maintenance protocol

**Figure 2:**
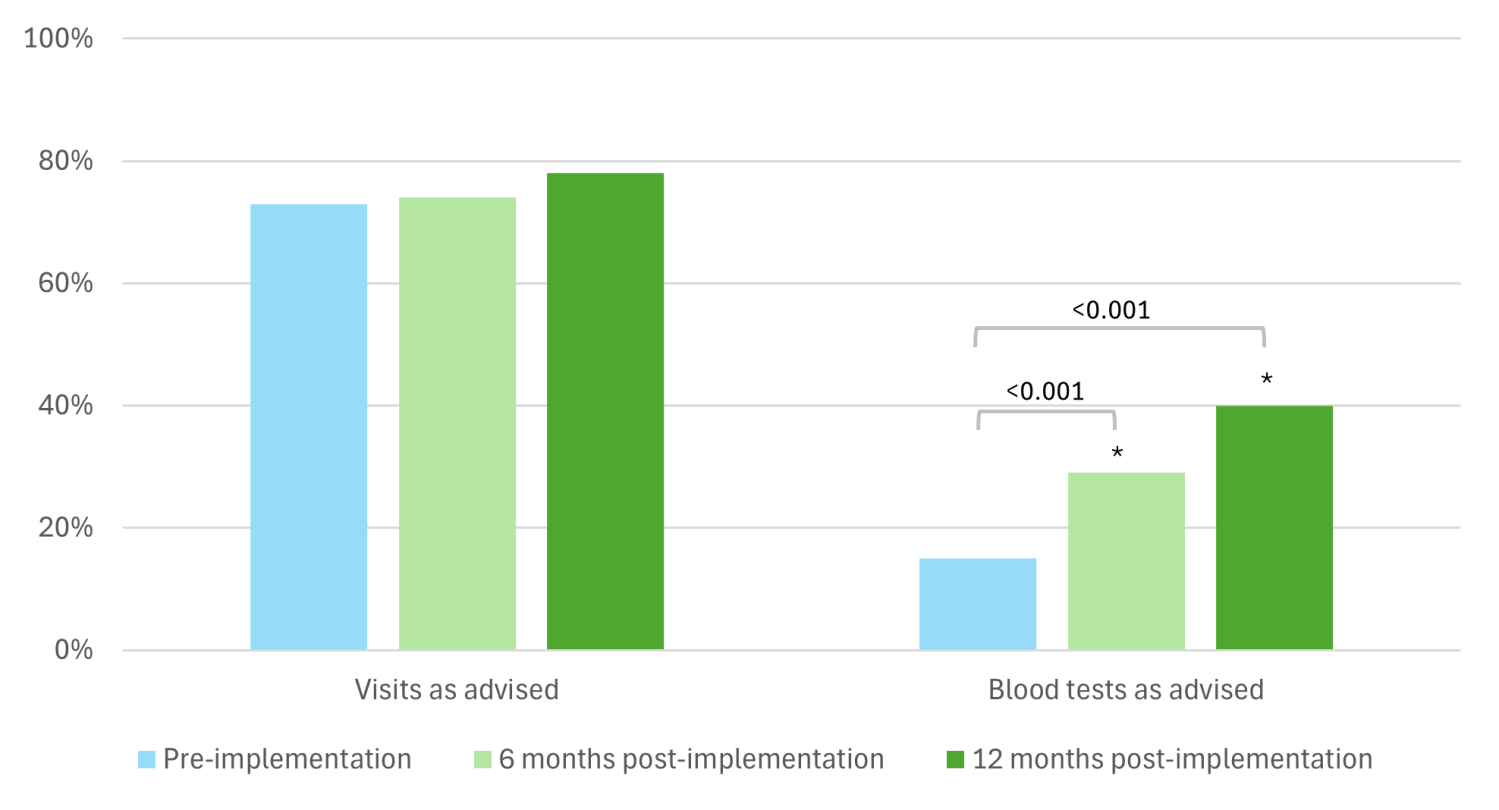
Adherence maintenance protocol. *Significant difference compared with the pre-implementation period.

For the maintenance protocol, adherence to ordering the advised blood tests increased significantly from 15% to 29% at 6 months and 40% at 12 months post implementation (p<0.001 for both) (figure 2).

#### Flare protocol

**Figure 3:**
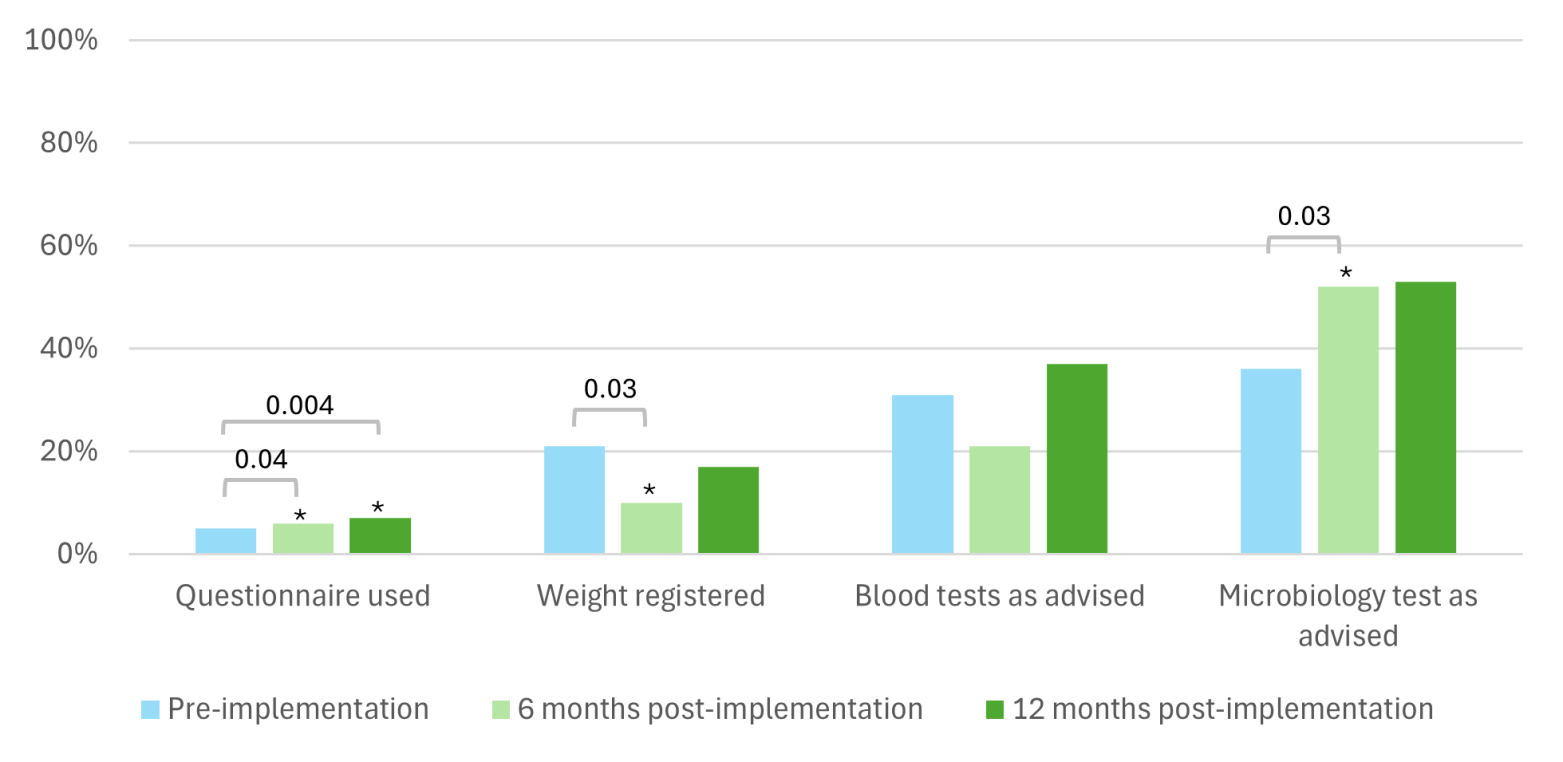
Adherence flare protocol. *Significant difference compared with the pre-implementation period.

Implementation of the flare protocol led to a modest yet statistically significant increase in the use of validated questionnaires for assessing disease activity, with the use increasing from 5% to 6% at 6 months post implementation (p=0.04), to 7% at 12 months post implementation (p=0.004) (figure 3). The percentage of weight registrations decreased from 21% to 10% (p=0.03) at 6 months, but did not change significantly at 12 months post implementation (17%, p=0.57). Adherence to ordering microbiology tests as advised improved after 6 months (p=0.03), but a significant increase was not sustained (p=0.09).

#### Annual outpatient visit protocol

**Figure 4:**
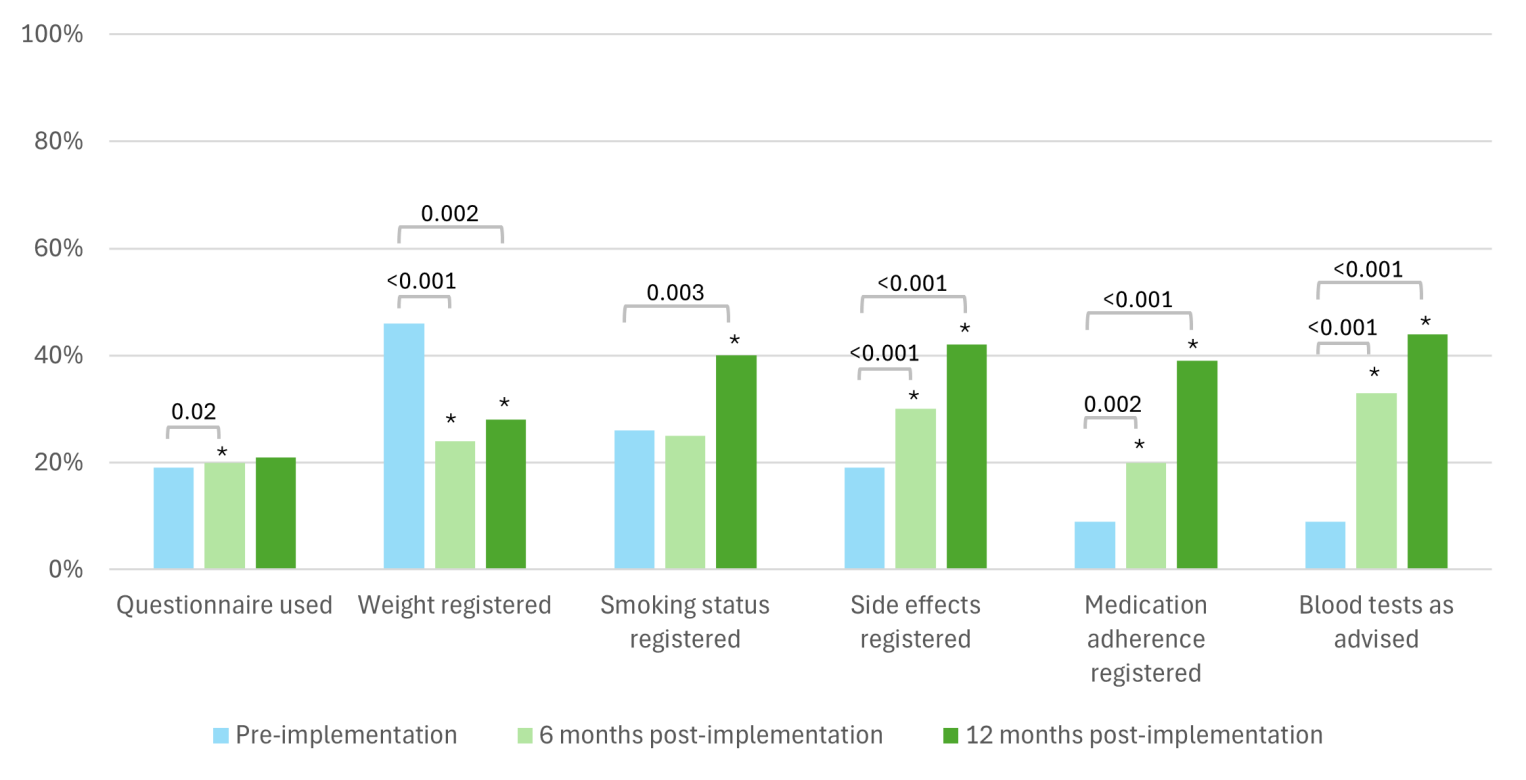
Adherence annual outpatient visit protocol. *Significant difference compared with the pre-implementation period.

During annual outpatient visits, the use of validated questionnaires to describe disease activity increased at 6 months post implementation (from 19% to 20%, p=0.02), but not at 12 months (p=0.30). Weight registration decreased from 46% to 24% at 6 months and 28% at 12 months (p<0.001 and p=0.002, respectively). Conversely, the registration of smoking status, side effects and medication adherence improved significantly. Smoking status registration increased to 40% at 12 months (p=0.003). Side effect registration increased to 30% at 6 months (p<0.001) and 42% at 12 months (p<0.001). Medication adherence registration also increased significantly to 20% at 6 months and 39% at 12 months post implementation (p<0.001 for both periods). Adherence to ordering blood tests as advised improved significantly to 33% at 6 months and 44% at 12 months post implementation (p<0.001 for both periods) (figure 4).

### Barriers and facilitators

**Figure 5:**
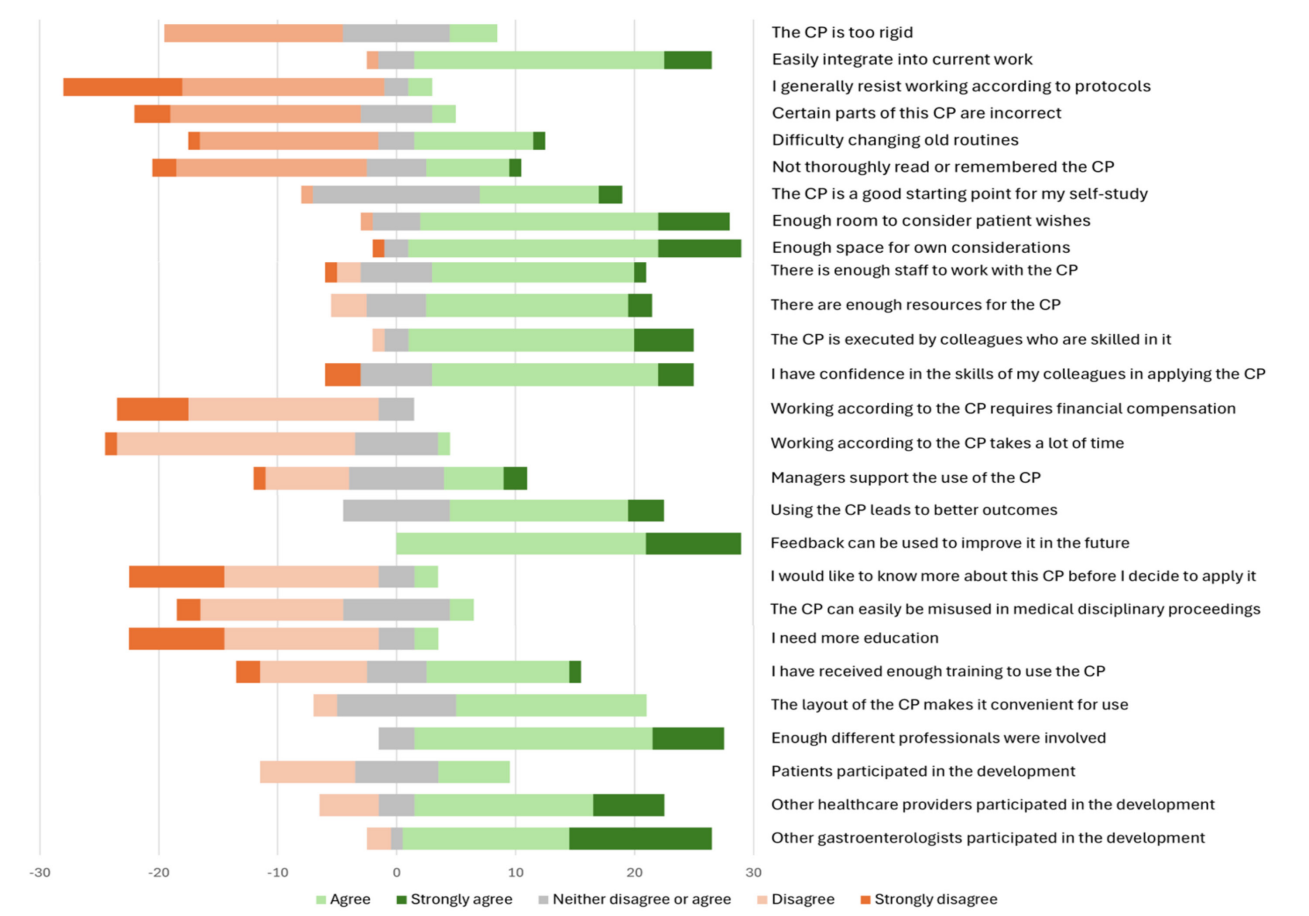
Survey results on constructs of the extended normalisation theory. CP, care pathway.

A total of 11 interviews were conducted with gastroenterologists, medical assistants, Information Technology (IT) personnel and IBD nurses. In addition, a questionnaire to assess barriers and facilitators in the implementation process was distributed to 85 HCPs, of whom 42 responded (49%) and 26 completed the questionnaire. Questionnaire results and the semistructured interviews were used to describe the four constructs of the eNPT (figure 5).

#### Capability

The identified elements related to the CP’s capability to be integrated into clinical practice were the potential of the CP to standardise care, to improve collaboration between HCPs, and that the CP was user-friendly. The CP enabled a more efficient and unified working method, reducing the risk of mistakes. While the potential for standardisation of care is seen as a major advantage, flexibility within the CP was highlighted as essential. The heterogeneity of IBD and the need to consider patient needs were identified as potential barriers, highlighting the need for HCPs to maintain the ability to personalise care when necessary. Most HCPs believed that the CP could be easily integrated into their daily practice. In all hospitals, the CP was integrated into the EHRs; however, the use was perceived as difficult. This could have been due to half of the respondents having difficulties changing their current working methods, not sufficiently knowing how to use the CP after the implementation in the EHR, or not thoroughly reading or remembering it to apply the CP in daily practice.

#### Capacity

HCPs generally agreed that there are enough resources and staff available to support the use of the CP. The implementation process in each hospital was supervised by one working group member, who worked with IT personnel to ensure implementation into the EHR. The involvement of different stakeholders with their specific knowledge was perceived as an important facilitator. However, lack of time, concerns about additional workload and that it could feel as an obligation emerged as barriers. Delays in implementing the CP in individual hospital IT systems were noted, as each hospital had its own infrastructure, requiring separate efforts to implement the CP.

#### Potential

Most respondents believed that the CP would lead to improved outcomes and reduced healthcare utilisation, reflecting positive perceptions of its potential. For some, the CP introduced only small adjustments to their current practice, making it challenging to recall the differences and implement the CP. Despite these barriers, enthusiasm and a shared interest in improving patient outcomes served as facilitators of the CP.

#### Contribution

A facilitator was the comment phase during the development, which allowed users to provide feedback on the CP before finalisation. The CP was perceived as evidence-based where available and flexible enough to allow for clinical judgement and patient preferences. Accessibility of the CP was another facilitator, with HCPs reporting that it was user-friendly and a useful tool for clinical guidance. However, there was no consensus on the sufficiency of training received; while half of the respondents felt under-trained, the majority indicated that additional education was unnecessary.

## Discussion

With this study, we aimed to investigate the adherence to a CP for the treatment of patients with IBD with advanced therapies, and identify barriers and facilitators for developing and implementing this CP. Adherence to the CP varied across protocols assessing different aspects of IBD treatment, and while it improved over a longer period following its implementation, further optimisation is still warranted. The most important facilitators were the potential to improve patient outcomes by standardising care, making collaboration between HCPs easier and ensuring that the CP is user-friendly for HCPs. Barriers included the complexity of the implementation of the CP in EHRs, the difficulty for HCPs to change their working routines and the heterogeneity of IBD.

Despite the CP being designed in accordance with current guidelines and in agreement with a working group, adherence rates remained low. Several factors may explain the results. One factor is the limited time available during outpatient visits to fully implement the CP into the work process of HCPs. Due to time constraints, HCPs may revert to old routines and skip tasks that seem more time-consuming. It is also plausible that these tasks were performed, but not documented in the EHR, creating discrepancies between actual tasks performed, care provided and the recorded information. The summary written in the EHR may omit information sought in this study. We attempted to mitigate this by incorporating standard texts HCPs could use in the EHR. These standard texts contained all the components of the protocols and could be filled in by the HCP. Despite this intended simplification, the system was perceived as difficult to use, which hindered its effective adoption.

Patient variability made the CP more difficult to apply in practice, as the heterogeneity of IBD can make it challenging to address all possible scenarios within a standardised CP.^29 30^ Efforts were made to incorporate this flexibility into the CP, allowing deviations where necessary. This flexibility was highly valued by users, as it allowed for more personalised care for the patients and autonomy for HCPs, which are often mentioned as barriers for implementing interventions.^30^ This indicates that the CP is mainly suited for standard situations as a reference tool, such as scheduling follow-up visits and blood tests when there are no additional comorbidities.

The difficulty in changing current working routines was a primary barrier identified in this study. The Transtheoretical Model suggests that behaviour change is not quick or decisive, but rather occurs through a cyclical process. In the initial precontemplation phase, people do not intend to act in the next 6 months, primarily seeing the disadvantages of changing their behaviour. In the following phases, individuals become increasingly prepared to change their behaviour until they eventually adopt the new behaviour.^31^ The CP was developed by a small group that recognised the benefits of changing their current practice. As a result, they were more willing to modify their practices and follow the CP as recommended. However, it is possible that not all colleagues were in this phase of the model and therefore less inclined to change their behaviour. This may also explain the observed increase in adherence towards the end of the post implementation year, as more HCPs may have progressed to a stage where they were ready to change their behaviour.

To encourage HCPs to progress through the phases of the Transtheoretical Model, it is important to create awareness of the CP and its benefits. This was attempted by offering a comment phase, where HCPs could provide feedback on the CP, allowing for further adjustments. This was also aimed to ensure agreement with the CP and to facilitate the implementation. In addition to the comment phase, there was a 3-month implementation period during which the CP was presented in each hospital. However, this one-time training may not have been sufficient to raise enough awareness. Respondents reported not remembering or reading the CP thoroughly enough and not feeling adequately trained to use it. Nevertheless, more than half also indicated that they did not wish to receive further training. This indicates that the HCPs might be in the precontemplation phase of the Transtheoretical Model. To move them into the next phase of the model, an alternative approach could be to avoid additional education about the CP and instead demonstrate its potential impact and the available support for its use, both key elements in behaviour change processes. An audit and feedback (A&F) meeting could be a good alternative for education. In such meetings, HCPs receive a summary of their adherence to the CP in comparison to their colleagues.^32^ In this study, HCPs might have been unaware of their suboptimal adherence and therefore lacked the motivation to change their current working method. A&F meetings can encourage them to change their practice and move to the next phase of the Transtheoretical Model. Additionally, these meetings could provide insight into which elements of the CP are most usable and what adaptations may be needed to improve uptake in clinical practice. In this study, HCPs’ perspectives on why adherence to certain components of the CP was low were not assessed. A&F meetings could address this gap by allowing HCPs to reflect on adherence patterns and to discuss which components are perceived as unclear, impractical or insufficiently supported in daily practice. Identifying these challenges would enhance the understanding of the CP’s usability and establish targets for refinement.

Our study has some limitations that need to be considered when interpreting these results. One limitation is that adherence data were collected from EHRs. Data are not based on standard measurements, but depend on the registration of HCPs. Registrations can vary due to differences in the use of the EHR, time constraints, or varying levels of detail considered necessary, leading to inconsistencies and variability. This may cause incomplete data and skewed representation of adherence, possibly under-reporting adherence. For measuring quality of care, complete and uniform EHR recording is important. It enables informed decision-making, reduces the risk of medical errors and facilitates communication between HCPs.^33^ A main reason for not using the provided standard texts and packages was the complexity to find them in the EHRs. When continuing to use the CP, emphasis should be placed on simplifying the use in EHRs, including reducing the number of standardised packages and texts.

Second, the potential association between adherence to the CP and patient outcomes was not assessed. While there is evidence that CPs can lead to better clinical outcomes through consistent care delivery, we cannot draw conclusions regarding the clinical impact of the CP.^34^ Evaluating patient outcomes was beyond our scope and should be addressed in future research.

Third, the patient perspective was not included. Interviews were conducted with gastroenterologists, medical assistants, IT personnel and IBD nurses, and questionnaires were sent to HCPs using the CP. We cannot assess how patients perceived the CP or the impact on their experience. Despite this, we believe the CP may not have negatively impacted the patient care experience. The CP represented a minor modification to the standard of care and allowed flexibility and deviations for individual patient needs. Moreover, patient representatives participated in the development of the CP, ensuring patient-centric design.

Another limitation is the small proportion that completed the questionnaires. This creates a risk of non-response bias, where respondents differ from those who did not. However, the mixed-methods approach provides a comprehensive understanding by combining objective adherence data with subjective insights into barriers and facilitators. This method identifies both adherence problems and underlying reasons, which is of importance for improving the CP in future research.

## Conclusions

A CP for the treatment of IBD with advanced therapies has been implemented to improve IBD care. Adherence to the CP appears to be challenging, partially contributed to the difficulty HCPs experience in changing their behaviour. However, the gradual improvement over time indicates that behavioural change is a process and increased familiarity may support its uptake. Facilitators such as the potential to improve patient outcomes through standardised care and the user-friendliness of the CP played a role in the adoption of this process. The suboptimal adherence might be due to a discrepancy between tasks performed and those registered in the EHR by HCPs. This could be improved by A&F meetings, which communicate current adherence and have the potential to identify reasons for low adherence to specific components of the CP. Future research should focus on how such meetings can be used to refine the CP and improve its uptake in daily clinical practice.

## Supplementary material

10.1136/bmjoq-2025-003583 online supplemental file 1

## Data Availability

Data are available upon reasonable request.
