# Supplementary material for: Adherence to a care pathway for inflammatory bowel disease in the southwest region of the Netherlands: results of a mixed-methods implementation study
Source: BMJ Open Qual. 2025 Oct 22;14(4):e003583. doi: 10.1136/bmjoq-2025-003583 (PMC12548584; doi:10.1136/bmjoq-2025-003583)
Supplement: online supplemental file 1 [file bmjoq-14-4-s001.docx]

Supplementary Files

Supplementary Table 1. Results per protocol

| **Outcome** | **Period** | **Percentage** | **P-value** |
| --- | --- | --- | --- |
| *Induction protocol* | | | |
| Weight registered | Pre-implementation | 31% |  |
|  | 6-months post-implementation | 21% | 0.27 |
|  | 12-months post-implementation | 50% | 0.38 |
| Screening repeated | Pre-implementation | 82% |  |
|  | 6-months post-implementation | 26% | <0.001 |
|  | 12-months post-implementation | 17% | <0.001 |
| Vaccinations registered | Pre-implementation | 15% |  |
|  | 6-months post-implementation | 10% | 0.42 |
|  | 12-months post-implementation | 33% | 0.26 |
| Patient information registered | Pre-implementation | 57% |  |
|  | 6-months post-implementation | 43% | <0.001 |
|  | 12-months post-implementation | 67% | <0.001 |
| Letter to GP | Pre-implementation | 31% |  |
|  | 6-months post-implementation | 45% | 0.16 |
|  | 12-months post-implementation | 67% | 0.04 |
| Visits as advised | Pre-implementation | 57% |  |
|  | 6-months post-implementation | 62% | 0.55 |
|  | 12-months post-implementation | 100% | - |
| Blood tests as advised | Pre-implementation | 10% |  |
|  | 6-months post-implementation | 43% | 0.42 |
|  | 12-months post-implementation | 33% | 0.50 |
| Combination therapy^a^ | Pre-implementation | 100% |  |
|  | 6-months post-implementation | 100% |  |
|  | 12-months post-implementation | 100% |  |
| *Maintenance protocol* | | | |
| Visits as advised | Pre-implementation | 73% |  |
|  | 6-months post-implementation | 74% | 0.13 |
|  | 12-months post-implementation | 78% | 0.15 |
| Blood tests as advised | Pre-implementation | 15% |  |
|  | 6-months post-implementation | 29% | <0.001 |
|  | 12-months post-implementation | 40% | <0.001 |
| *Flare protocol* | | | |
| Questionnaire used | Pre-implementation | 5% |  |
|  | 6-months post-implementation | 6% | 0.04 |
|  | 12-months post-implementation | 7% | 0.004 |
| Weight registered | Pre-implementation | 21% |  |
|  | 6-months post-implementation | 10% | 0.03 |
|  | 12-months post-implementation | 17% | 0.57 |
| Blood tests as advised | Pre-implementation | 31% |  |
|  | 6-months post-implementation | 21% | 0.14 |
|  | 12-months post-implementation | 37% | 0.48 |
| Microbiology test as advised | Pre-implementation | 36% |  |
|  | 6-months post-implementation | 52% | 0.03 |
|  | 12-months post-implementation | 53% | 0.09 |
| *Annual visit protocol* | | | |
| Questionnaire used | Pre-implementation | 19% |  |
|  | 6-months post-implementation | 20% | 0.02 |
|  | 12-months post-implementation | 21% | 0.30 |
| Weight registered | Pre-implementation | 46% |  |
|  | 6-months post-implementation | 24% | <0.001 |
|  | 12-months post-implementation | 28% | 0.002 |
| Smoking status registered | Pre-implementation | 26% |  |
|  | 6-months post-implementation | 25% | 0.90 |
|  | 12-months post-implementation | 40% | 0.003 |
| Side effects registered | Pre-implementation | 19% |  |
|  | 6-months post-implementation | 30% | <0.001 |
|  | 12-months post-implementation | 42% | <0.001 |
| Medication adherence registered | Pre-implementation | 9% |  |
|  | 6-months post-implementation | 20% | 0.002 |
|  | 12-months post-implementation | 39% | <0.001 |
| Blood tests as advised | Pre-implementation | 9% |  |
|  | 6-months post-implementation | 33% | <0.001 |
|  | 12-months post-implementation | 44% | <0.001 |

Abbreviations: GP; general practitioner.

^a^When starting infliximab
